# Potential Use of Seaweed Bioactive Compounds in Skincare—A Review

**DOI:** 10.3390/md17120688

**Published:** 2019-12-06

**Authors:** Valentina Jesumani, Hong Du, Muhammad Aslam, Pengbing Pei, Nan Huang

**Affiliations:** 1 Guangdong Provincial Key Laboratory of Marine Biotechnology College of Sciences, Shantou University, Shantou 515063, China; tina@stu.edu.cn (V.J.); 18nhuang@stu.edu.cn (N.H.); 2 Faculty of Marine Sciences, Lasbela University, Uthal 90950, Pakistan

**Keywords:** seaweeds, hyperpigmentation, skin aging, skincare, photo-protection

## Abstract

Modern lifestyles have developed new attention on appearance and personal care which attract a huge number of consumers towards cosmetic products. The demand for a skincare product with natural ingredients is rapidly increasing. Seaweeds are major resources for in-demand active compounds with a wide variety of applications. The use of seaweed-derived ingredients in cosmetic products has increased in recent years as many scientific studies have proved the potential skincare properties of seaweed bioactive compounds. This review emphasizes possible skincare properties of seaweed bioactive compounds. The review outlines the mechanism involved in skin problems including hyperpigmentation, premature skin aging, and acne in the first part while the second part focuses on the promising application of seaweeds in skin protection by highlighting the bioactive compound responsible for their bioactivity.

## 1. Introduction

Cosmetics are the materials used to enhance or alter the function and appearance of the skin and hair [1]. Kligman created the term “cosmeceutical” to hightlight cosmetic products that can combine the use of both cosmetic and pharmaceutical uses [2]. Cosmeceuticals are often used in dermatology to enhance the skin tone, skin glow, and provide anti-aging benefits [3]. The cosmeceutical industries are most fascinating, profitable, and constantly growing in the world economy. According to reports, an average woman spends $15,000 on beauty products in her lifetime [4]. The cosmetics industry has predicted an annual gross revenue of US $170 billion according to the financial exploration stated by a French-based company, Eurostaf [5]. In 2016, the European cosmetics market was top in the world, esteemed at €77 billion in a wholesale rate, trailed by the US and Brazil [4]. The global beauty market stated that the cosmetic industry will continue to develop due to the growth of the middle class in many developing countries [6]. Based on this encouraging future of the cosmetics industry, many cosmetic products without any side effects have been developed to satisfy the customers’ needs. Currently, many synthetic chemicals have been used in cosmetic products, many of them did not get synthetic customer satisfaction due to high cost and unsafe nature in terms of side effects. For example, chemicals like hydroquinone, arbutin, and kojic acid are being used as a skin whitening agent, but they are reported to be unstable and they also cause dermatitis and induce cancer [7,8,9]. Thus, in recent years, the demand for cosmetic products that containing natural ingredients is rapidly expanding. The advantages of natural ingredients are environmentally friendliness, fewer side effects, and safe use [1,10]. Hence, Cosmeceutical industries are persistently seeking active compounds from natural sources. From this perspective, the marine environment provides numerous marine organisms, including seaweeds with potential bioactive compounds. Seaweeds are rich in bioactive compounds that could be exploited as functional ingredients for cosmetic applications [11]. This review focusses on the cosmetic properties of seaweed bioactive compounds and provides an overview of skin problems and the potential of seaweed bioactive compounds against skin problems.

## 2. Structure of Skin

The skin is the major organ in the human body. Generally, the skin can be divided into epidermis, dermis, and subcutaneous tissue. The epidermis is the uppermost layer of the skin. It has three types of cells—namely keratinocytes, melanocytes, and Langerhans cells. Keratinocytes are made up of keratin, which on maturation lose water and move up to the uppermost layer of the epidermis called the ‘stratum corneum’ [12]. The next collection of cells present in the epidermis are melanocytes, the cells that produce melanin, the pigment accountable for skin tone and color. Langerhans cells inhibit the penetration of unwanted foreign materials into the skin. The condition of the epidermis defines the freshness and youthfulness of your skin. The middle layer of the skin is the dermis [13]. Collagen and elastin are the main components of the extracellular matrix (ECM), covering about 90% of the dermis, which are cross-linked and provide support for the skin. Hence, the dermis is responsible for the structural support and elasticity of the skin. Collagen is found in the extracellular matrix of all animal bodies [14]. Hyaluronic acid (HA) is also a main constituent of the dermis. HA plays an important role in moisture absorption and moisture retention [12]. Subcutaneous tissue, which is the third layer located under the dermis, is comprised of connective tissue and fat. The loss of subcutaneous tissue due to age will also lead to facial sagging and wrinkles.

## 3. UV Induced Skin Damage

The ultraviolet (UV) radiation from the sun extends the earth in a significant amount. UV-C (100–290 nm) is mostly filtered by the atmosphere, but UVA (320–400 nm) and UVB (290–320 nm) rays extend the skin and cause suntan, wrinkles, etc. [15]. UV radiation induces the production of reactive oxygen species (ROS) and also depletes the antioxidant enzymes [16]. These ROS can lead to skin disorders such as hyperpigmentation (dark spots), premature aging, dryness, etc. [17,18].

### 3.1. Hyperpigmentation

Hyperpigmentation is usually an inoffensive form in which spots of the skin become darker in color than the regular surrounding skin. The overproduction and accumulation of melanin pigment resulted in a change in skin color. Melanogenesis is controlled by an enzyme such as tyrosinase, a glycoprotein [19] present in the membrane of the melanosome which catalyzes the conversion of l-tyrosine to melanin [20]. Melanogenesis is regulated by maturation and translocation of tyrosinase. The translocation of tyrosinases is regulated by the presence of specific carbohydrate moieties [21].

**Figure 1:**
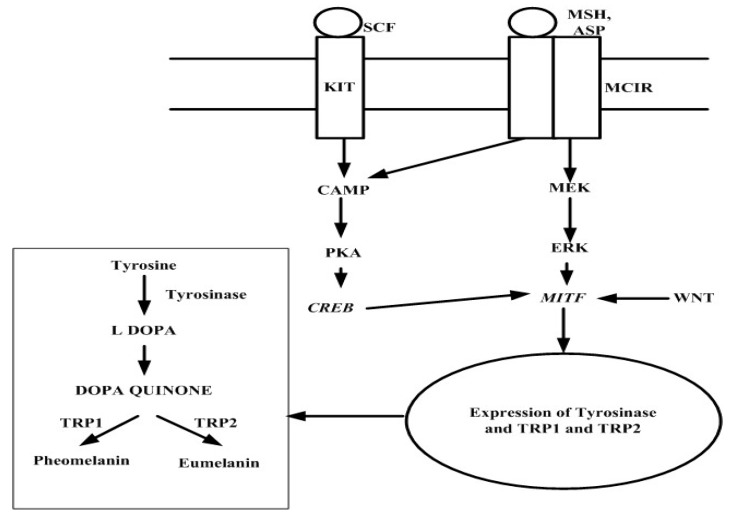
Signaling pathways involved in melanin synthesis. Tyrosinase-related protein (TRP), microphthalmia-associated transcription factor (MITF), adenosine 3′,5′-cyclic monophosphate (cyclic AMP) (cAMP), cAMP response element-binding (CREB), extracellular receptor kinase (ERK), melanocortin 1 receptor (MC1R), wingless-related integration site (Wnt), α-melanocyte-stimulating hormone (MSH), agonist stimulating protein (ASP), and stem cell factor (SCF).

Two types of melanin are synthesized within melanosomes: eumelanin and pheomelanin. The pathway in which melanogenesis occurs is presented in Figure 1. The enzymes such as tyrosinase and tyrosinase related protein (TRP-1 and 2) are produced by the phosphorylation of MITF, which is activated by several signaling pathways such as cAMP, ERK, and Wnt pathways. These signaling pathways are upregulated by the upstream of the receptor such as KIT (ligand SCF) and MC1R (ligand α-MSH, ACTH, and ASP). The KIT receptor activates the cAMP pathway and MC1R activates both cAMP and ERK pathway which further phosphorylates the MITF. This leads to the expression of tyrosinase-related enzymes which further mediates the production of melanin [22,23,24,25,26]. The skin under UV generates the reactive oxygen species (ROS) that activate the α-MSH and MC1R and enhances the production of tyrosinase that leads to the excess generation of melanin [27,28].

### 3.2. Skin Aging

**Figure 2:**
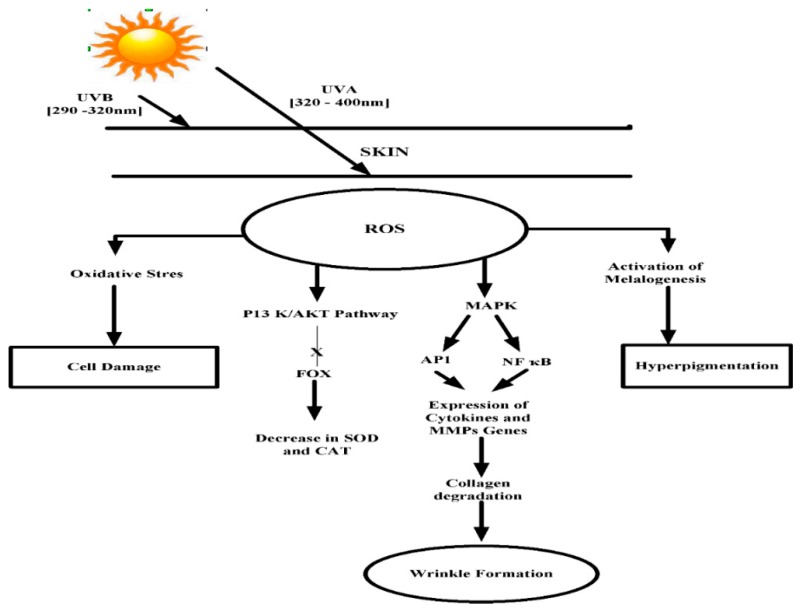
UV induced signaling pathway involved in premature skin aging. Mitogen-activated protein kinase (MAPK), matrix metalloproteinase (MMP), nuclear factor kappa-light-chain-enhancer of activated B cells (NF-κB), activator protein 1 (AP-1).

Skin aging is a complex process that occurs in all living beings that caused by two factors. One is intrinsic in which aging is caused by genetics [29]. The latter one is an extrinsic factor, in which aging occurs due to the exposure of skin to the ultraviolet rays. This type of aging is called photo-aging or premature aging [30]. Reactive oxygen species (ROS) play a key role in skin aging. ROS triggers the various growth factors and cytokine receptors which further stimulate mitogen-activated protein kinase (MAPK) signal transduction and P13/AKT pathway. The AKT pathway inactivates the FoxO which suppresses the expression of antioxidant enzymes in the cell. MAPK upregulates activator protein-1 (AP-1) and NF-κB in the nucleus. The induction of AP-1 gives rise to the MMP expressions [31,32] (Figure 2). MMPs are a collection of zinc-containing extracellular proteinases that degrade the extracellular components, such as collagen and elastic fibers, inducing wrinkle formation [31,32,33]. ROS also activates the expression of the hyaluronidase enzyme that degrades hyaluronic acid. Hyaluronic acid is present in extracellular matrix, absorbing and retaining water molecules and helping to keep the skin smooth, moist, and lubricated [34,35,36].

## 4. Bacteria-Induced Skin Damage-Acne Vulgaris

Acne vulgaris is a prevalent, chronic skin disorder which affects most of the adult and leads to scar marks. Acne vulgaris is a formation of lesions and prevalently caused by *Propionibacterium acnes*. Acne is spread by enzymes such as lipase, protease, hyaluronidase, and acid phosphatase produced by *P. acnes* [37]. The infection of *P. acne* triggers the immune response by the release of cytokine (IL-12 and IL-8) and the antimicrobial peptide (β-defensins) expression [38]. IL-8 stimulates neutrophils movement which leads to the formation of acne lesions and pus. Neutrophils consequently produce free radicals for killing the bacteria. This excess production of free radicals leads to the development of the inflammatory responses [39]. *Staphylococcus aureus* and *Staphylococcus epidermidis* are also the normal flora of human skin may also cause acne inflammatory response but are less significant than *P. acnes* in this process [40].

## 5. Seaweeds a Potential Source in the Cosmetic Industry

**Figure 3:**
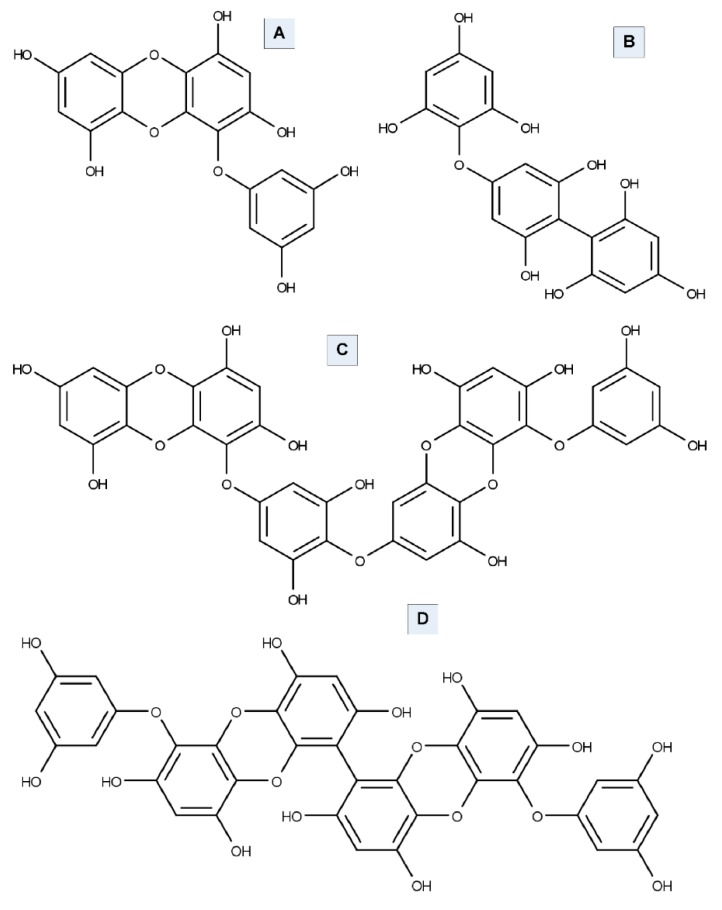

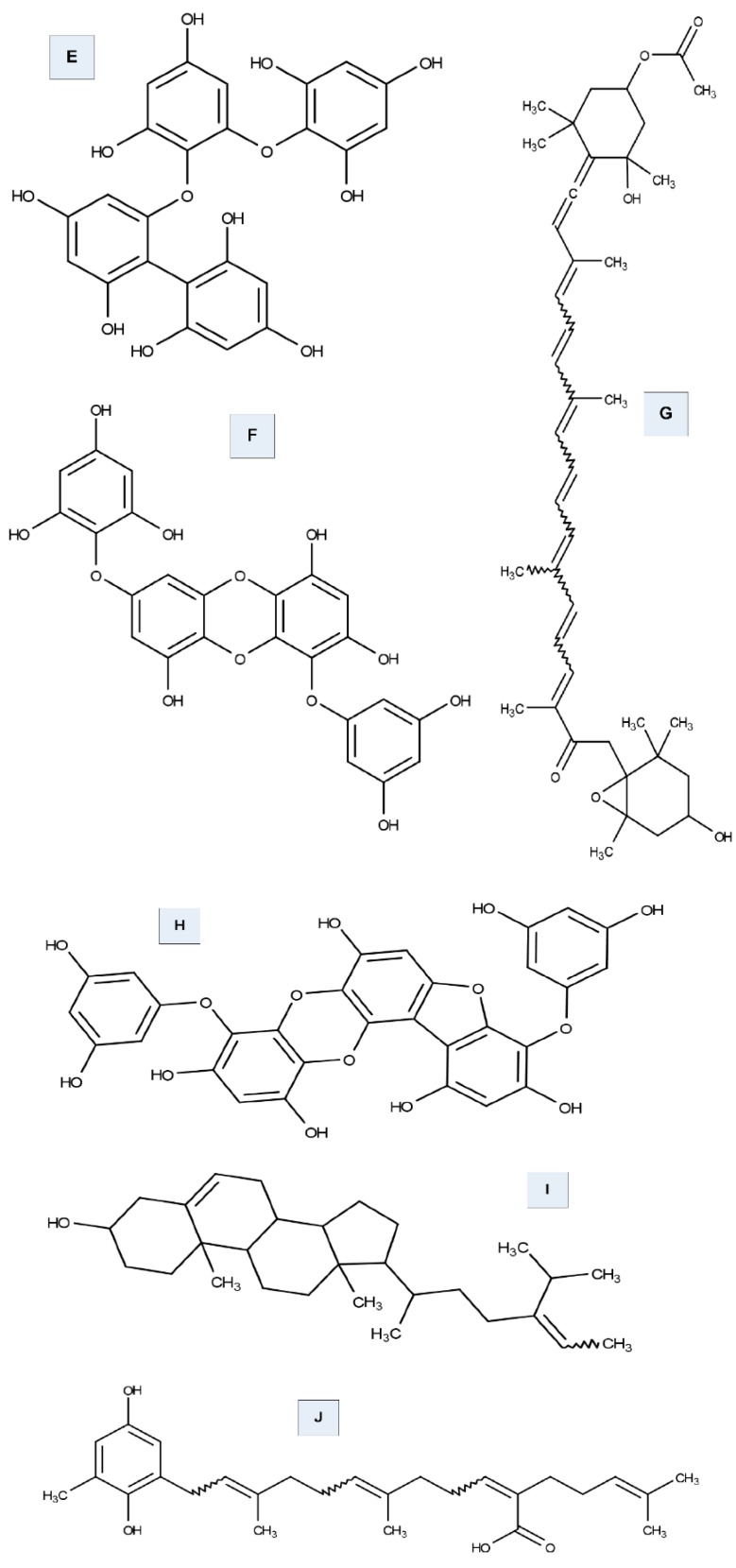

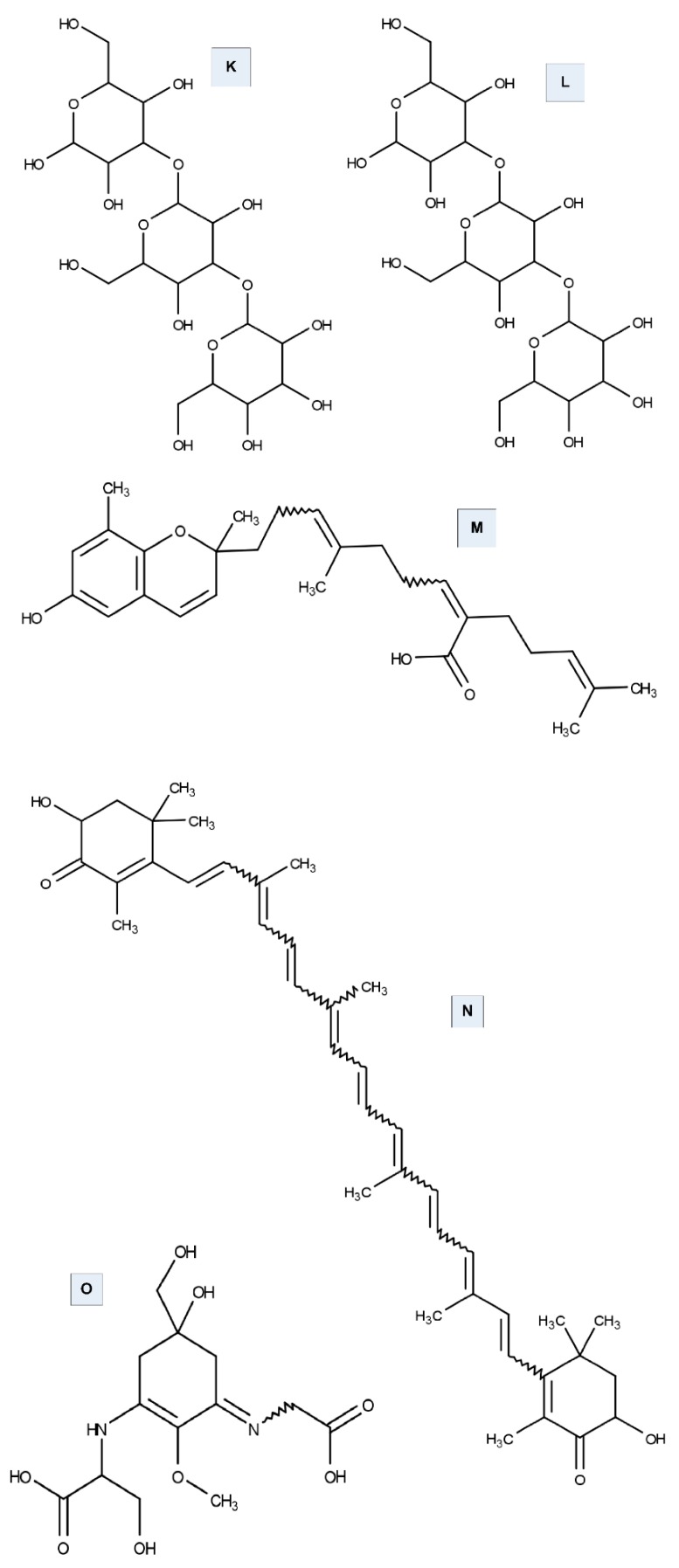
Seaweed bioactive compounds with skincare potentials. (**A**) Eckol; (**B**) Fucophloroethol; (**C**) Dieckol; (**D**) 6,6 Bieckol; (**E**) Fucodiphloroethol G; (**F**) 7-phloroeckol; (**G**) Fucoxanthin; (**H**) phlorofucofuroeckol; (**I**) Fucosterol; (**J**) Sargahydroquinoic acid; (**K**) Laminarin; (**L**) Porphyra 334, (**M**) Sargachromenol; (**N**) Astaxanthin; (**O**) Shinorine [3,22,42,47,57].

Nowadays People prefer cosmetic products that have natural ingredients than chemical ones. As the products with natural ingredients are safe to use without any side effects, many consumers go in search of natural products to keep themselves look young with healthy skin. Due to this, the cosmetic industry has also focussed on the ingredients that are derived from natural resources like plant, algae, microbes, and their metabolites. The marine world is extremely demanding for a wide variety of species with multiple bioactive compounds. Macroalgae are major resources for the active compound with a wide variety of applications in many fields (Figure 3) [16].

Macroalgae or seaweeds are the aquatic, photosynthetic organisms taxonomically categorized as algae, and they divided into three groups based on their pigment, the Rhodophyceae (red algae), Phaeophyceae (brown algae), and Chlorophyceae (green algae). Marine algae are considered as sea vegetables which are also used for consumption. Since ancient times seaweeds are also used as an alternative medicine for skin-related diseases. Many studies revealed the potentiality of seaweeds and their major role in antioxidant, antitumor, anti-inflammatory, anti-lipedemic, anti-microbial, and also their anti-allergic properties. Wide applications of seaweeds are based on the valuable bioactive compounds and potent bioactivity. In addition, the compounds derived from marine algae have been given considerable importance in developing a cosmeceutical product [41]. Seaweed compounds—including phenolic compounds, polysaccharides, pigments, PUFA, sterols, proteins, peptides, and mycosporine-like amino acid (MAA)—exhibited a wide range of bioactivity that can be used as active ingredients in cosmetic products (Figure 3) [7,42]. Phenolic compounds are the water-soluble secondary metabolites that have numerous biological activities [43]. It is a diverse group of compounds and the common structural features shared by all the phenol groups. Based on the number of substituents, phenolic compounds can be divided into simple phenols or polyphenols. Flavonoids and gallic acid are the building blocks of polyphenols. Phenolic compounds from seaweeds, like *Ecklonia cava* Kjellman and *Ishige okamurae* Yendo, are proven to have many bioactivities—including anti-oxidant, anti-microbial, anti-inflammatory, anti-cancer, etc. Antioxidant activity of seaweeds is mainly due to the presence of phenolic compounds [43,44]. Among the many phenolic compounds extracted from seaweeds, phlorotannins from brown seaweed are the most important secondary metabolites, with a wide range of functional bioactivity [45]. Phlorotannins are phloroglucinol-based polyphenols found in Marine brown algae. Phloroglucinol units linked to each other in various ways to form phlorotannins [46]. Marine brown algae such as *Ecklonia cava* Kjellman, *E. stolonifera* Okamura, *E. kurome* Okamura, *Ishige okamurae* Yendo, *Hizikia fusiformis* (Harvey) Okamura, *Eisenia bicyclis (Kjellman) Setchell Undaria pinnatifida* (Harvey) Suringar, *Sargassum thunbergii* (Mertens ex Roth) Kuntze, and *Laminaria japonica.* Areschoug have been studied the biological activity of phlorotannins [47,48]. Phlorotannins are well known for their wide-ranging applications which include anti-melanogenesis, anti-aging, and antioxidant [49,50,51,52]. As a result of the bioactivities, the application of phlorotannins on pharmaceutical, nutraceutical, and cosmeceutical advances [43,53,54].

Polysaccharides are the most important compounds present in seaweeds and are well documented for its biological activity. The green seaweed-like Ulva has the high content of polysaccharide comprises of 65% of dry weight. The other seaweeds that have a large amount of polysaccharide are Ascophyllum, Porphyra, and Palmaria species. The important polysaccharides are ulvan from green seaweeds, fucoidan, alginate, and laminarin from brown seaweeds, agar, and carrageenan from red seaweeds. In this, agar and alginate are used widely in the food industry as thickening and gelling agents. Fucoidan, ulvan, and carrageenan are sulfated polysaccharides that have wide application in many fields. Among these polysaccharides, the fucoidan from brown seaweed has been studied enormously for their bioactivity including antioxidant, anticancer, antimicrobial, hyperlipedemic, anti-inflammatory, etc. [4]. In recent days, many studies recommend the use of polysaccharide as an active ingredient in cosmetic formulations. Polysaccharides have a huge number of cosmetic roles such as hair conditioners, moisturizers, emulsifiers, wound-healing agents, and as a thickening agent [55,56].

Proteins are macromolecules made up of one or more amino acids. Seaweeds are a good source of amino acid. Amino acids are one of the important constitutes of natural moisturizing factor which prevents the water loss in the skin [57]. Seaweeds have amino acids, such as alanine, proline, arginine, serine, histidine, and tyrosine. Palmaria and Porphyra have the maximum amount of arginine, which is considered a natural moisturizing factor that can be used in cosmetic products. Mycosporine-like amino acids are water-soluble low molecular weight molecules. They are categorized by cyclohexane joined with nitrogen as a substitute for amino acid, amino alcohol, or amino group [57]. For seaweeds exposed to extreme stress including UV radiation, Mycosporine-like amino acids defend seaweed from UV radiation and act as a potent photo protector candidate. It also involved in radical scavenging and DNA repair systems. Hence, they have received more attention as UV protection and antioxidant agents in the cosmetic industry [3,16,58]. Furthermore, in recent years, peptides have drawn attention in the field of skincare due to their binding specificity to the target cells and their ability to change the physiological functions in the skin. Bioactivity depends on the composition of amino acids.

Macroalgae contain a large variety of pigments which absorb the light for photosynthesis. The green algae contain the pigment similar to the plants namely chlorophylls a, b, and carotenoids. The red algae have photosynthetic pigments such as chlorophyll a and the phycobilins such as R-phycocyanin and R-phycoerythrin and carotenoids, mostly β-carotene, lutein, and zeaxanthin. The brown algae pigments include the chlorophylls a and c, fucoxanthin, and carotenoids. The pigment act as a shield to the cells from UV irradiation [59]. Seaweeds are an important source of vitamin A, vitamin B, vitamin C, vitamin D, and vitamin E which are widely used in skincare.

The lipid content of seaweeds is generally low and less than 4% of the dried mass, whereas *Sargassum kjellmaniamum* Yendo contains more than 6% [60]. Lipids such as essential fatty acid, glycolipids, sterols, triglycerides, and phospholipids are found in seaweeds. Polyunsaturated fatty acid (PUFA) present in seaweeds is higher than in terrestrial plants. Seaweed fatty acids have anti-allergic and anti-inflammatory activities and also act as an emollient that protects the skin from water loss [61].

## 6. Skincare Application of Seaweeds

In recent years, seaweeds have been most desirable source of research for their bioactivity and bioactive compounds like polyphenols, fucoidan, phlorotannins, carotenoids, etc. Beauty care products have been focused on compounds with potential antioxidant activity, MMPs, and tyrosinase inhibitory activity in order to reduce ROS caused by UV radiation and also to delay skin aging.

### 6.1. Tyrosinase Inhibition Activity of Seaweed

Tyrosinase is the enzyme that catalyzes the synthesis of melanin, a pigment that is responsible for skin color. Hyperpigmentation is caused due to the abnormal accumulation of melanin pigments in the skin. Overexposure to UV rays induces abnormal melanin synthesis which results in skin pigmentation. Tyrosinase inhibitors may act as a candidate for the control of hyperpigmentation or skin whitening as the tyrosinase catalyzes the melanogenesis [62]. The search for natural tyrosinase inhibitors becomes a great interest for non-toxic and active skin whitening ingredients. Hence, skin whitening agents derived from seaweeds could be advantageous for the cosmetic industry. Researchers screened various seaweed extracts for tyrosinase inhibition activity and found that *Ishige okamurae* Yendo, *Endarachne binghamiae* J.Agardh, *Schizymenia dubyi (Chauvin ex Duby) J.Agardh*, *Ecklonia cava*, *E. stolonifera Okamura*, and *Sargassum silquastrum* (Mertens ex Turner) C.Agardh showed profound tyrosinase activity and significantly reduced the content of the melanin [63,64,65]. *S. polycystum hexane extract* had no inhibitory activity on mushroom tyrosinase. However, it showed potential activity on cellular tyrosinase inhibition when examined on cellular tyrosinase [66]. Dieckol is a phlorotannin derivative isolated from *E. stolonifera* showed the tyrosinase inhibition activity with the IC50 of 2.16 μg/mL [67,68]. Fucoxanthin is a carotenoid present in the seaweed exhibits tyrosinase inhibition activity when treated orally and also applied topically in UVB-induced guinea pig [69]. Many studies proved that sulfated polysaccharide, fucoidan extracted from Fucus sp., Sargassum sp., and Laminaria sp. can also be used as a promising tyrosinase inhibitor [70,71,72]. Fucoidan, the polysaccharide extracted from the brown seaweed such as *Chnoospora minima* (Hering) Papenfuss and *Sargassum polycystum* C.Agardh inhibit the activity of collagenase, elastase and also tyrosinase [70]. Tyrosinase activity was increased by the low molecular weight fucoidan extracted from *Sargassum fusiforme* (Harvey) Setchell [73]. Park et al. [74] were also demonstrated the increased inhibitory activity in low molecular weight fucoidan in a melanoma cell.

Several signaling pathways involved in melanin synthesis. The cAMP pathway is one of the prime regulatory mechanism which increases the expression of microphthalmia-associated transcription factor -MITF. MITF regulates the expression of tyrosinase, tyrosinase-related protein 1,2 which is required for melanogenesis. Ethyl acetate fraction of *Leathesia difformis* Areschoug showed the effect on melanin synthesis and cellular tyrosinase activity by downregulating the CREB, PKA, and cAMP pathways [75]. ERK pathway involves in anti-melanogenesis. The phosphorylation of ERK degrades the MITF which leads to the suppression of melanin synthesis [23]. Fucoidan plays a major role in the anti-melanogenesis by ERK phosphorylation [76]. Another study showed the inhibitory activity of fucoidan on cellular melanin and tyrosinase but in contrast, it lacks the inhibitory activity on the expression of TRP1, TRP2, and MITF [71]. Sargahydroquinoic acid, sargachromenol, and sargaquinoic acid from *S. serratifolium* (C.Agardh) C.Agardh decreased the α-MSH-activated melanogenesis in melanoma cells through the inhibition of CREB signaling pathways without affecting ERK pathway [77]. Sulfated galactans, the polysaccharide from *G. fisheri*, showed no potential inhibition on tyrosinase activity and it proved to be suppressed the activity of tyrosinase by downregulating the MITF, TRP1,2, and tyrosinase mRNA expression, which was concluded by RT-PCR and ELISA [78].

### 6.2. Collagenase and Elastase Inhibition Activity of Seaweed

The MMPs are a family of degradative enzymes particularly collagenase which is responsible for the degradation of skin matrix especially collagen due to which occurs the skin sagging. The same way enzyme elastase degrades the elastin. This process leads to wrinkles. The compound that inhibits collagenase and elastase activity might act as an active ingredient in an anti-aging product. Overexposure of UV produces ROS which activates the mitogen-activated protein kinases followed by the phosphorylation of transcription factor activator protein1 results in the upregulation of MMPs.

Seaweed polysaccharides play a major role in inhibiting collagenase and elastase activity. Sulfated polysaccharides from *Sargassum fusiforme* (Harvey). Setchell potentially inhibited the activity of intracellular collagenase and elastase by regulating the NF-κB, AP-1, and MAPKs pathways in HDF cells radiated by UVB [79]. Fucoidans isolated from the *Chnoospora minima* (Hering) Papenfuss and *Sargassum polycystum* C.Agardh showed elastase and collagenase inhibition in a dose-dependent manner [70]. Fucoidan inhibited the expression of MMP 1 in UVB -induced dermal fibroblast cells in a dose-dependent manner. It suppressed the expression of MMP by inhibiting the ERK pathway and reduced the expression of MMP1 mRNA. Furthermore, Fucoidan also inhibited the activity of the MMP1 promoter and increased the expression of Type 1 procollagen synthesis [80,81].

Ryu et al. [82] proved that methanol extracts of *Corallina pilulifera* J.V.Lamouroux that are rich in phenolic content inhibited the MMP 2,9 expressions in a dose-dependent manner in UV-induced dermal fibroblast cells. Phlorotannin extracted from *Eisenia bicyclis* (Kjellman) Setchell, *Ecklonia cava* Kjellman, and *E. stolonifera* Okamura strongly inhibit the MMP1 expression. Similarly, phlorotannin from *E. cava* inhibit the expression of MMP 2,9 and also reduced the activity of MMPs at 10 µg/mL. Eckol, dieckol, dioxinodehydroeckol, and bieckol are responsible for the inhibition of MMPs in human dermal fibroblast cells. This previous study also suggested that these phlorotannin derivatives inhibited the expression of NF-kappa B and AP-1 reporter resulting in the suppression of MMP expression [51,83]. The results of all these studies suggest that phlorotannin may act as an active ingredient in preventing photoaging of the skin.

The peptides, namely PYP1-5 and Porphyra 334 from *Porphyra yezoensis* f. coreana Ueda, increased the production of elastin and collagen and decrease the expression of MMP protein [84]. PYP1-5 induced the collagen synthesis by initiate the TGF-b/Smad signaling pathway by increasing the expression of TIMP-1,2 and TGF-b1 protein expression [85]. Likewise, Sargachromanol extracted from *S. horneri* (Turner) C.Agardh also activated the TIMP1,2 and downregulate the expression of MMP [86]. The sterol compound, fucosterol from marine brown algae also enhanced the production of type I procollagen and suppressed the expression of MMPs in human keratinocytes cell by deactivating the MAPK pathway [59]. All these studies revealed the potential protection of seaweed bio compounds towards UVA-induced collagen degradation.

### 6.3. Hyaluronidase Inhibition

Hyaluronidase is an enzyme that degrades the hyaluronic acid present in the extracellular matrix which results in the skin aging process. Very few studies have been focused on Hyaluronidase inhibition. Phlorotannins derivatives—such as fucophloroethol, fucodiphloroethol, fucotriphloroethol, 7-phloroeckol, phlorofucofuroeckol, and bieckol/dieckol extracted from *Cystoseira nodicaulis* (Withering) M.Roberts exhibited the hyaluronidase activity with the IC50 of 0.73 mg/mL and also proved that the higher molecular weight displayed the strongest activity [87]. Phlorotannins derivatives—such as dieckol, eckol, bieckol, and phlorofucofuroeckol A—extracted from *Eisenia bicyclis* (Kjellman) Setchell and *Ecklonia kurome* Okamura exhibited potent inhibition towards hyaluronidase. Among these phlorotannin derivatives, bieckol exhibited the strongest inhibition with an IC50 value of 40 μM [88].

### 6.4. Photoprotection Ability

When the skin is exposed to UV radiation, UV rays penetrate into dermis and epidermis and induce the production of ROS which cause damage to DNA. This results in hyperpigmentation, premature aging, sunburn, skin cancer, etc. The extensive use of photoprotection products will help to get rid of the effect caused by the sun UV rays. The macroalgae are exposed to extreme conditions such as UV radiation and it produces the ROS. The seaweeds produce many secondary metabolites that play as an antioxidant that helps to combat those ROS. These antioxidant substances included pigments like fucoxanthin, carotenoids, mycosporine-like amino acids (MAA), and phenols such as phlorotannins and scytonemins [89,90]. These bioactive components are capable of absorbing the UV radiation and keep the human fibroblast cells from UV-induced aging. These secondary metabolites that act as UV filters/sunscreen with antioxidant activity can be extensively used in cosmetic products. Polysaccharides such as fucoidan, laminarin, and alginate extracted from brown algae like *Fucus vesiculosus var. alternans C.Agardh*, Sargassum sp, *Turbinaria conoides f. laticuspidata W.R.Taylor*, possessed potent anti-oxidative activity [89].

Cardozo et al. [91] studied the MAA from the red algae, *Gracilaria birdiae* E.M.Plastino & E.C.Oliveira, *G. domingensis* (Kützing) Sonder ex Dickie, and *G. tenuistipitata* C.F.Chang & B.-M.Xia which exhibited the photoprotection activity. Heo et al. [92] studied the photoprotection ability of fucoxanthin on UV induced human fibroblast cells and showed to significantly inhibit the cell damage at 61.24% at 250 μM. Fucoxanthin successfully suppressed the cell damage and apoptotic stimulation induced by UV-B. Fucoxanthin extracted from *Sargassum fusiforme* (Harvey) Setchell and *S.saliquastrum* (Mertens ex Turner) C.Agardh exhibited strong antioxidant activity against DPPH and hydrogen peroxide. Urikura et al. [93] reported that fucoxanthin reduced UV induced ROS in the hairless mice and also suppress the MMP expression.

A red pigment, astaxanthin, exhibited strong antioxidant activity and protects from peroxidation by scavenging the radicals. The activity may be due to the presence of conjugated polyene and terminal ring moieties of astaxanthin help to trap the radicals and therefore exhibit potent antioxidative and photoprotective agents. It also blocked cytokine production. The topical application also demonstrated the photoprotection effect against the cell damage caused by UVB radiation [94]. Lyons et al. [95] studied the photoprotection exhibited by the algal extract that contains astaxanthin by reducing DNA damage and also conserve the cellular antioxidant enzymes in human cells irradiated by UVA. Tetraprenyltoluquinol chromane meroterpenoid extracted from *Sargassum muticum* (Yendo) Fensholt showed potent photoprotection against UV-A radiation and also inhibit the accumulation of intracellular ROS in human dermal fibroblast cells [96].

Phlorotannins extracted from *E. cava* and *E. stolonifera* also provided photoprotection towards UVB rays by reducing the cell damage caused by UVB radiation which is measured by comet assay. It also showed the inhibition against UVB induced oxidative damage with antioxidant activities and also upgrading in cell viability [63,68]. Phlorotannins extracted from *Halidrys siliquosa* (Linnaeus) Lyngbye proved the sunscreen ability based on the sun protection factor and UV-A protection factor. It also was shown to exhibit strong antioxidant activity and proved to have the ability to kill bacteria [97]. Vo et al. [98] isolated fucofuroeckol-A, which exhibited the photoprotection activity against damage caused by UVB radiation. The aqueous extracts of *Hydropuntia cornea* (J.Agardh) M.J.Wynne and *Gracilariopsis longissima* (S.G.Gmelin) Steentoft, L.M.Irvine & Farnham exhibited a photo-protective activity with the sun protection factor of 7.5 and 4.8. The MAA Porphyra-334 and Shinorine were extracted from *Porphyra rosengurttii* J.Coll & J.Cox tested for their photo-protective activity and photo-stability without producing any free radicals. The formulated product of these two demonstrated wide-ranging protection against UV. MAA can also absorb UV light and also act as a sunscreen [99,100]. Porphyra-334 from the *Porphyra umbilicalis* Kützing reduced the intracellular ROS induced by UV-A radiation and also suppressed the expression of MMPs. It also acts as a better UV filter compared to synthetic sunscreens [84,101].

### 6.5. Moisture Retention Ability

Maintaining moisture in the skin is important to skincare and it improves the skin texture and state, i.e., young and healthy. Extract from *Undaria pinnatifida* (Harvey) Suringar, *Codium tomentosum* Stackhouse, *Durvillea antarctica* (Chamisso) Hariot, *Cladosiphon okamuranus* Tokida, *A. nodosum* (Linnaeus) Le Jolis, *Pediastrum duplex* Meyen, and *Polysiphonia lanosa* (Linnaeus). Tandy exhibited skin hydrating properties and protects the skin from dryness. Polysaccharides have maximum water holding capacity which can act as a humectant and moisturizer in cosmetic industry. Polysaccharides from *Laminaria japonica* Areschoug were shown to have greater hydrating and moisturizing effects than hyaluronic acid. The formulation prepared by incorporating *Laminaria japonica* extract shown to improve the skin moisture [3,4,6]. Shao et al. [102] isolated sulfated polysaccharide from the green algae *Ulva fasciata* Delile displayed a higher capability both in the moisture-absorption and moisture retention for 96 h when compared with glycerol. Wang et al. [103] extracted the polysaccharide from *Saccharina japonica* (Areschoug) C.E.Lane, C.Mayes, Druehl & G.W.Saunders; *Porphyra haitanensis* T.J.Chang & B.F.Zheng; *Codium fragile* (Suringar) Hariot; *Enteromorpha linza* (Linnaeus) J.Agardh and *Bryopsis plumose* (Hudson) C.Agardh and studied for the moisture absorption and retention. The authors also proved that the sulfate content and molecular weight plays a major role in the moisture-holding capacity [103].

### 6.6. Antimicrobial Activity

Antimicrobial property of the seaweeds can be used in cosmetic products as a preservative that could delay the shelf life of the cosmetic product by killing the microorganisms especially fungi that could spoil the product. Seaweeds exhibited antifungal activity for possible use in substituting chemical preservations. Seaweeds such as *Sargassum vulgare* C.Agardh, *Colpomenia sinuosa* (Mertens ex Roth) Derbès & Solier, *Dictyopteris membranacea* Batters, *Cystoseira barbata* (Stackhouse) C.Agardh, and *Dictyota* dichotoma (Hudson) J.V.Lamouroux, showed the strongest antifungal effect against *Cladosporium cladosporioides*, *Alternaria alternata*, *Fusarium oxysporum*, *Aspergillus niger*, *Epicoccum nigrum*, *A.ochraceus*, *Penicillium citrinum*, and *A. flavus* [104]. Extract from *Halimeda tuna* (J.Ellis & Solander) J.V.Lamouroux also showed the antifungal activity against *Candida albicans*, *Aspergillus niger*, *A. flavus*, *Alternaria*, *Trichophyton rubrum*, *Epidermophyton floccossum*, *T. mentagrophytes*, and Penicillium sp. [44]. Saidani et al. [105] studied the antifungal activity of seaweed, in which the seaweed, *Rhodomela confervoides* (Hudson) P.C.Silva reported for the strongest inhibition against *Candida albicans* and *Mucor ramaniannus* and the seaweed, *Padina pavonica* (Linnaeus) Thivy against the *Candida albicans*. Phlorotannin derivative, Dieckol from *E.cava* showed the antifungal activity with the MIC of 200 μM against *Trichophyton rubrum* [106]. Alghazeer et al. [107] screened the 19 seaweed extracts and tested them for their antibacterial activity. Their data revealed that all the extracts showed inhibition against gram-positive and gram-negative bacteria including *E. coli*, *Staphylococcus aureus*, and *S. epidermis*. Among the 19 species the brown algae *Cystoseira crinita* Duby showed the strongest antibacterial activity. *Ulva rigidis* also showed the strongest inhibition against *S. aureus* and *Escherichia coli* [108]. These studies confirmed the role of seaweeds and their extract as a preservative.

The skin may also be contaminated by some microorganisms which could create skin problems that can be overcome by the antimicrobial potential of the seaweeds derived biological compounds. *Propionibacterium acnes, Staphylococcus aureus*, and *S. epidermis* are some of the normal microflora present in the skin. *P. acnes* is the main inducers of acne. *S. aureus* and *S. epidermis* are harmless microflora, but it can enter the skin epidermis through the wound and cause infection by secreting the toxins [109]. This leads to pimples, abscesses, and also blisters. Therefore, the antimicrobial potential of seaweed could be used effectively in cosmetic formulations in the prevention of skin acne [4,109]. Ruxton and Jenkins [110] discussed the anti-acne activity of seaweed oligosaccharide-zinc complex extracted from *Laminaria digitata* (Hudson) J.V.Lamouroux which also reduces the signs of acne by reducing the sebum production. Ethyl acetate extraction of *Fucus evanescens* C.Agardh showed antibacterial ability against methicillin-resistant *S. aureus* and *P. acnes* [111]. Choi et al. [112] screened 57 seaweed species for the antimicrobial activity against *P. acnes* in which 15 species exhibited the antiacne activity. The methanol extract of *E. cava*, *E. kurome*, *Ishige sinicola* (Setchell & N.L.Gardner) Chihara, and *Symphyocladia latiuscula* (Harvey) Yamada showed potent activity with the maximum MIC of 0.31 mg/mL. Phlorotannins isolated from *E. bicyclis* showed effective inhibitory activity against human acne producing bacteria such as *P. acnes*, *Staphylococcus aureus*, and *S. epidermidis* [113]. Carrageenan extracted from red algae of the genus Corallina inhibited the bacteria *S. epidermidis* with a MIC of 0.325 mg/mL, whereas sulfated galactan from Corallina showed the bactericidal activity against *Enterococcus faecalis* and *S. epidermidis* [114]. These studies defined that the seaweed compound can act as an ingredient for the anti-acne product due to its inhibitory activity against *P. acnes*, *S. aureus*, and *S. epidermidis*.

## 7. Conclusions

Due to the overexposure of human skin to several environmental stress—such as UV and pollution—it increases the production of ROS that leads to many skin related problems such as hyperpigmentation, premature aging, etc. The seaweeds in the marine environment have the biosynthesis of secondary metabolites for its survival under stress conditions. These biologically active components present in the seaweeds paves the way to be used as an active ingredient in the cosmetic industries due to their potent skin protection ability. The active components from the seaweeds could be used as an antioxidant, antibacterial whitening agent, anti-aging, and anti-acne, and also for moisturization in cosmetic industries.

## 8. Future Perspectives

This review examines the potentiality of seaweed-derived compounds in applications to combat skin whitening and aging in cosmetic industries. Though most of the seaweeds are studied for its cosmetic properties, still many species are not explored. Hence, the standardization of cost-effective and efficient methods to extract the bioactive compounds with higher productivity and activity is in demand. In addition to efficiency, the molecular mechanism of their activity and safety concerns of these compounds are very significant for future challenges in the cosmetics industry.

## Abbreviations

ECM	Extracellular matrix
HA	Hyaluronic acid
ROS	Reactive oxygen species
TRP	Tyrosinase-related protein
MITF	Microphthalmia-associated transcription factor
cAMP	Adenosine 3’,5’-cyclic monophosphate (cyclic AMP)
CREB	cAMP response element-binding
ERK	Extracellular receptor kinase
ASP	Agonist stimulating protein
SCF	Stem cell factor
Wnt	Wingless-related integration site
MC1R	Melanocortin 1 receptor
α-MSH	α-Melanocyte-stimulating hormone
MAPK	Mitogen-activated protein kinase
MMP	Matrix metalloproteinase
NF-κB	Nuclear factor kappa-light-chain-enhancer of activated B cells
AP-1	Activator protein 1
IL-12 and IL-8	Interleukin 12
TLR2	Toll-like receptor 2
MAA	Mycosporine-like amino acid
PUFA	Polyunsaturated fatty acid
